# α-synuclein in the pathophysiology of Alzheimer’s disease

**DOI:** 10.1186/s13024-019-0320-x

**Published:** 2019-06-11

**Authors:** Daniel Twohig, Henrietta M. Nielsen

**Affiliations:** Department of Biochemistry and Biophysics, Stockholm University, Svante Arrhenius Väg 16B, 10691 Stockholm, Sweden

**Keywords:** α-synuclein, Alzheimer’s disease, Lewy pathology, tau

## Abstract

The Alzheimer’s disease (AD) afflicted brain is neuropathologically defined by extracellular amyloid-β (Aβ) plaques and intraneuronal neurofibrillary tangles composed of hyperphosphorylated tau protein. However, accumulating evidence suggests that the presynaptic protein α-synuclein (αSyn), mainly associated with synucleinopathies like Parkinson’s disease (PD), dementia with Lewy bodies (DLB) and multiple system atrophy (MSA), is involved in the pathophysiology of AD. Lewy-related pathology (LRP), primarily comprised of αSyn, is present in a majority of autopsied AD brains, and higher levels of αSyn in the cerebrospinal fluid (CSF) of patients with mild cognitive impairment (MCI) and AD have been linked to cognitive decline. Recent studies also suggest that the asymptomatic accumulation of Aβ plaques is associated with higher CSF αSyn levels in subjects at risk of sporadic AD and in individuals carrying autosomal dominant AD mutations. Experimental evidence has further linked αSyn mainly to tau hyperphosphorylation, but also to the pathological actions of Aβ and the *APOEε*4 allele, the latter being a major genetic risk factor for both AD and DLB. In this review, we provide a summary of the current evidence proposing an involvement of αSyn either as an active or passive player in the pathophysiological ensemble of AD, and furthermore describe in detail the current knowledge of αSyn structure and inferred function.

## Background

Alzheimer’s disease (AD) is currently untreatable, the leading cause of dementia and the most prevalent neurodegenerative disorder worldwide [1–3]. The disease afflicts tens of millions of patients and burdens scores of caregivers [4]. The vast majority of AD cases arise sporadically either in the form of late-onset AD in individuals ≥65 years old (~90% of patients), or as early-onset AD when patients become AD symptomatic between ~45-65 years old (~6-16% of patients)[1, 5]. A small group of patients (~1%) have early-onset autosomal dominant AD (ADAD) caused by mutations in either the *PSEN1*, *PSEN2* or *APP* genes. The gradual cognitive decline and eventual dementia due to AD is thought to relate to the increased deposition of insoluble protein aggregates in the form of extracellular senile plaques enriched with aggregated amyloid-β peptide (Aβ), and intracellular neurofibrillary tangles (NFTs) primarily comprised of hyperphosphorylated microtubule-associated protein tau (tau). However, there is significant clinical and pathological heterogeneity between AD patients at all stages of AD pathogenesis. Advances in functional neuroimaging have made ante-mortem identification of aggregated forms of Aβ and tau possible, and along with fluid biomarkers, specifically cerebrospinal fluid (CSF) protein levels of Aβ residues 1-42 (Aβ_1-42_), total tau (t-tau) and phosphorylated tau (pThr181) (p-tau), offer a high degree of sensitivity and specificity for detecting pre-clinical, prodromal and probable AD [6–9].

Amongst the repertoire of genetic and lifestyle related risk factors associated with sporadic AD [10–12], the most significant risk factors are a family history of sporadic AD, increasing age (≥65 years old) and the presence of the epsilon-4 (ε4) allele of the *APOE* gene (*APOE*ε4) [1]. After the age of 65 the risk for developing AD doubles every five years eventually effecting >25% of people ≥85 years old [13], while individuals who are *APOE*ε4 heterozygous or homozygous have a 3-15-fold increased risk of AD, respectively [14–16]. Moreover, it has been recently shown that the increased risk associated with the *APOE*ε4 allele and AD symptom onset is more pronounced in early onset sporadic AD patients and females. A non-proportional clinical decline was found in both sexes, however, this began at age 75 in females compared to age 80 in males [17].

Interestingly, the *APOE* gene variants not only affect the risk of AD but also the closely related disorder dementia with Lewy bodies (DLB). Similar to the case with AD, the *APOE*ε4 variant significantly increases the risk of DLB, whereas the *APOE*ε2 variant appears to be protective against both disorders [16]. Dementia with Lewy bodies, as the name implies, is characterized by the occurrence of intracellular Lewy bodies and Lewy neurites which contain high concentrations of aggregated α-synuclein (αSyn). These patients frequently exhibit AD pathology, including Aβ plaques and to some degree tau pathology [18] which ante-mortem can be reflected in an AD-like CSF AD biomarker profile [19–21]. The implications of co-occurring AD pathology in DLB are currently unknown. Importantly, co-morbid αSyn pathology has been reported in more than half of all autopsy-confirmed AD cases [22–24], and more than 90% of patients with ADAD due to mutations in the *PSEN1* gene exhibit αSyn pathology specifically in the amygdala [25]. Studies from our own laboratory and those of others have provided results supporting the notion that αSyn may be involved in the earliest stages of AD development, reflected in altered CSF αSyn levels and associations with brain Aβ-plaque deposition in both sporadic AD and ADAD cases [26, 27]. Importantly, our recent study described a novel link between the *APOE*ε4 risk allele, CSF αSyn levels and Aβ deposition at the very early stages of AD development [27]. Therefore, we speculate that αSyn indeed contributes to the pathophysiology of AD as an underappreciated member of the pathological AD ensemble. In the current report we summarize the evidence provided by clinical and experimental studies linking αSyn to the pathophysiology of AD.

## Alzheimer’s disease pathophysiology and diagnosis

Alzheimer’s disease is an untreatable neurodegenerative disorder clinically defined by gradual cognitive decline with impairments in executive function, language, praxis, and visual processing that eventually lead to dementia. Pathologically the symptoms can be correlated to the loss of synapses, neurons, neuropil and an overall reduction of grey matter [28, 29]. The bulk of AD cases arise sporadically after the age of 65 years while roughly 10% [30] of AD diagnoses occur before the age of 65 years (early-onset AD). Many AD patients first present with subjective memory complaints then progress, at variable rates, to mild cognitive impairment (MCI) before ultimately being diagnosed with probable AD [31–33]. A small subpopulation (<1%) of early-onset AD patients harbor highly penetrant autosomal dominant mutations in the *APP* gene which encodes the amyloid precursor protein (APP), or within the *PSEN1* and *PSEN2* genes encoding two independent catalytic subunits of the APP protease γ-secretase, specifically presenilin-1 (PS1) and presenilin-2 (PS2) [34]. Consequently, the direct link between APP, APP processing and AD formed the basis of the widely accepted amyloid cascade hypothesis which postulates that a protracted asymptomatic disease phase with well-defined spatiotemporal accumulations of pathological Aβ plaques in the temporal lobe, frontal lobe and limbic system precedes widespread neocortical and limbic NFTs, symptom onset, cognitive decline and dementia [34]. Therapeutics designed to reduce Aβ aggregate load or inhibit either β- or γ-secretase have exhibited some promising results in animal models, but have not translated successfully into clinical trials, exemplified by well-publicized failures of costly Phase-II and –III clinical trials. Subsequently, the amyloid cascade hypothesis has become target of a fierce debate [35–37]. As a result, many proponents of the amyloid cascade hypothesis have now shifted their focus upstream to examine the toxicity of soluble mono- and multimeric forms of Aβ and/or tau due to a considerable body of evidence supporting this theory [38, 39].

An accurate clinical diagnosis of probable AD is problematic due to the considerable symptomatic heterogeneity between dementia patients [40]. As a result, a definite diagnosis of AD is still only possible upon post-mortem examination [41]. With the aim to improve diagnostics, a concerted push to define robust biomarker-based diagnostic frameworks for pre-clinical and probable AD is currently underway [42, 43]. For example, ante-mortem diagnosis of probable AD may be greatly improved by advances in neuroimaging and CSF biomarker analysis [6]. Structural imaging using magnetic resonance imaging (MRI) combined with functional PET radiotracers such as glucose analogues, Aβ plaque and NFT-specific ligands, have provided seminal insights into the molecular and physical changes occurring from the early to advanced stages of AD [44]. Accumulations of Aβ aggregates can begin years to decades prior to the onset of symptoms but appear to plateau once individuals become symptomatic [6]. The proposed spread of Aβ pathology tends to follow a spatiotemporal pattern beginning in the neocortex and subsequently spreading to the allocortex, basal ganglia, midbrain, and finally appearing in the pons and cerebellum [45]**.** Tau pathology also begins to develop during the pre-clinical phase of AD but much later relative to Aβ plaques. The occurrence of NFT continues to increase once patients are symptomatic and correlate well with AD symptom onset and cognitive decline [46]. Generally, AD NFT pathology is believed to start in the transentorhinal region and propagate to the entorhinal region, hippocampus and temporal neocortex, superior temporal neocortex, and eventually the entirety of the neocortex [45].

With brain PET imaging techniques for tau and Aβ plaque mainly still under development and/or refinement, the most utilized and informative AD biomarkers are found primarily in the CSF, and to a lesser extent in blood [47]. In CSF, the established AD biomarker panel supporting a diagnosis of probable AD are reductions in Aβ_1-42_ or the Aβ_1-42_/Aβ_1-40_ ratio, and increased levels of total tau and phosphorylated (pThr181) tau. This combination of CSF biomarkers is also sensitive enough to detect asymptomatic pre-clinical AD patients and can predict the decline from MCI to AD [48]. Increased levels of CSF total tau and phosphorylated tau in particular show a robust positive correlation to cognitive decline, a finding also verified by tau PET imaging [49].

## Post-mortem observations of α-synuclein in the AD brain

In addition to what we know as classical AD brain pathology including Aβ and tau lesions, numerous studies have also documented the occurrence of co-morbid αSyn or Lewy-related pathology (LRP) in more than 50% of autopsy-confirmed AD brains [22–24], please see Table 1 for an overview of the literature. Initially, Ueda and coworkers identified and described a non-Aβ peptide fragment, which we now know to be the non-amyloid-β component (NAC) region of αSyn [54], enriched in Aβ plaques isolated from a single patient. These results were later confirmed in a larger cohort of autopsy-confirmed AD cases [56]. Upon closer examination it indeed has turned out that a substantial proportion of AD patients examined at autopsy exhibited LRP in the neocortex [52, 57], limbic system [22–24, 61–63, 65], and to a lesser extent in the substantia nigra [23, 65]. Co-occurring LRP may relate to visual hallucinations [63], extrapyramidal symptoms [51, 53, 72] and a shorter more aggressive disease course [59]. Although fewer post-mortem studies have investigated the occurrence of LRP in ADAD cohorts, two studies have shown that individuals with *PSEN1* mutations have increased, yet heterogeneous limbic LRP [22, 25]. In a small, but notable ADAD kindred study, three related family members carrying the S170F *PSEN1* mutation had widespread limbic, neocortical and brainstem LRP, dementia onset during the third decade of life, severe symptoms and death by the fourth to fifth decade of life [64].

**Table 1:** Post-mortem evidence demonstrating involvement of α-synuclein in AD pathophysiology

Type of Analysis	Cohort	Findings	Citation (Year)
• Brain tissue	• *N*=225 - AD (*n*=150) - NC (*n*=75)	• 25% of AD cases had LBs (versus 5% of NCs) - 11% of which had cortical LBs - 3% of which had neocortical LBs and where re-classified as DLB - LBs primarily in SNc, substantia innominata and locus coeruleus • Inverse relationship between LBs and tau pathology	• Bergeron and Pollanen (1989) [50]
• Brain tissue • Clinicopathological correlations	• *N*=36 - AD (*n*=23) - AD-LBV (*n*=13)	• AD-LBV patients had: - more pronounced cognitive and movement symptoms versus AD - less tau pathology - increased spongiform vacuolization of the medial temporal lobe - increased neurodegeneration of the SNc, substantia innominata, and locus coeruleus	• Hansen et al. (1990) [51]
• Brain tissue	• *N*=147 AD	• 25% where “Aβ-plaque only” AD - of which, 75% had LRP • 28% were AD-LBV - of which 66% were “Aβ-plaque only” AD	• Hansen et al. (1993) [52]
• Brain tissue	• *N*=16 - AD (*n*=8) - AD-LBV (*n*=8)	• AD-LBV patients had: - higher incidence of Parkinsonism - increased frontal lobe atrophy - reduced frontal lobe and limbic tau-tangles - increased neuronal loss within the SNc and the nucleus basalis of Meynert which correlated with reduced cognitive function	• Förstl et al. (1993) [53]
• Brain tissue	• *N*=1x human cortical AD Aβ-plaque • *N*=1x AD brain section sections	• Found a novel 35-amino acid sequence in an Aβ-plaque - named it the “non-amyloid component (NAC) of Aβ-plaques” (NAC) - used novel antibodies to detect the NAC in hippocampal neuronal soma and neurites in AD brain sections	• Uéda et al. (1993) [54]
• Brain tissue	• *N*=137 - probable AD or AD-LBV	• 30 % of cohort confirmed as AD-LBV • In AD-LBV, not AD, *APOEe4* associated with enhanced NFTs	• Hansen et al. (1994) [55]
• Brain tissue	• *N*=75 - AD (*n*=68) - NC (*n*=7)	• NAC immunoreactivity found: - in 35% of diffuse Aβ-plaques - in 55% of mature Aβ plaques - primarily in the core of Aβ-plaques	• Masliah et al. (1996) [56]
• Brain tissue • Clinicopathological correlations	• *N*=50 - AD Mild (*n*=4) Moderate (*n*=16) Severe (*n*=23) - NC (*n*=7)	• NAC immunoreactivity: - increased in frontal cortex of mild AD individuals - versus all other groups - correlated negatively with NFTs - did not correlate with Aβ-plaques correlated negatively with cognitive decline	• Iwai et al. (1996) [57]
• Brain tissue	• *N*=74 - ADAD *PSEN1* (*n*=57) *APP* (*n*=9) *PSEN2* (*n*=8)	• LRP immunoreactivity: - found in 22% of all ADAD brains - found in 63% (12/19) of ADAD amygdala samples - frequently alongside tau-tangles in amygdala - not influenced by *APOEε4*	• Lippa et al. (1998) [58]
• Database search	• *N*=188 - AD (*n*=148) - AD-LBV (*n*=40)	• Compared to AD, AD-LBV patients had: - more rapid cognitive decline - shorter survival time after symptom onset	• Olichney et al. (1998) [59]
• Brain tissue	• *N*=20 - Down’s syndrome with AD (*n*=16) without AD (*n*=4)	• LRP in 50% of Down’s syndrome brains with AD - primarily in the amygdala	• Lippa et al. (1999) [60]
• Brain tissue	• *N*=145 - AD	• LRP in 61% of AD brains - primarily in the amygdala and entorhinal cortex - rarely in the SNc - frequently alongside NFTs	• R.L. Hamilton (2000) [23]
• Brain tissue	• *N*=25 - AD (*n*=23) - Down’s syndrome with AD (*n*=2)	• LRP immunoreactivity: - in 43% of AD and 100% of Down’s syndrome - primarily in the amygdala - frequently alongside NFTs	• Marui et al. (2000) [61]
• Brain tissue	• *N*=35 - AD (*n*=24) - AD-LBV (*n*=8) - NC (*n*=3)	• In AD-LBV patients LRP: - primarily in hippocampus, less frequently in the frontal cortex - often co-localized with Aβ-plaques in dystrophic neurites	• Wirths et al. (2000) [62]
• Brain tissue	• *N*=27 - AD	• LRP found in ~50% cases - most frequently in the amygdala - most non-LB LRP found in hippocampus - frequently co-localized with NFTs	• Arai et al. (2001) [24]
• Brain tissue	*N*=60 - AD (*n*=17) - DLB (*n*=34) - PD (*n*=9)	• In AD cases: - LBs, not LNs, frequently found in amygdala - no limbic or neocortical LRP - 95% co-occurrence of LBs and NFTs in amygdala	• Iseki et al. (2004) [63]
• Brain tissue • Clinicopathological correlations	*N*=3 - ADAD *PSEN1* (S170F) mutation carrying family members	• Rapid decline in third decade of life • Severe symptoms, i.e. myoclonus, rigidity and seizures • Death in fourth decade or early fifth decade of life • Florid LBs in neocortex, limbic system and brainstem	• Snider et al. (2005) [64]
• Brain tissue	*N*=28 - AD	• LBs found in: - hippocampus (54% of cases) - amygdala (47%) - SNc (42%) - entorhinal cortex (33%) • Correlation between LRP and Aβ pathology • No correlation between LRP and NFTs	• Mikolaenko et al. (2005) [65]
• Brain tissue	*N*= 39 - ADAD 14 *PSEN1* (*n*=25) *PSEN2* (N141I) (*n*=14)	• In *PSEN1* cases: - 96% had amygdala LBs - frequent neocortical and amygdala LBs versus *PSEN2* • In both *PSEN1* and *PSEN2* cases: - significant variability of Lewy body pathology between family members with same ADAD mutation.	• Leverenz et al. 2006 [25]
• Brain tissue • Clinicopathological correlations	*N*=347 - AD	- In total 43% had some extent of LRP - 25% diagnosed as AD-LBV - 24% AD cases had amygdala LRP with sparse LRP in other limbic regions - LBs and NFT frequently co-localize in same soma - αSyn and tau frequently co-localize in the same lesion - no clinical difference in AD with amygdala LRP versus AD cases	• Uchikado et al. 2006 [66]
• Brain tissue	*N*=12 - AD (*n*=4), - DLB (*n*=4) - NC (*n*=4)	• In AD and DLB, but not NC brain samples, αSyn monomers, dimers, trimers and pentamers co-immunoprecipitated with Aβ monomers	• Tsigelny et al. 2008 [67]
• CSF	*N*=325 - AD (*n*=131), - DLB (*n*=40), - FTD (39), - VsD (*n*=39), - NC (*n*=112)	• CSF αSyn levels did not differ between dementia groups	• Spies et al. 2009 [68]
• Brain tissue	• *N*=84 - AD (*n*=24) - MCI (*n*=34) - NC (*n*=26)	• In AD brains without LRP there was a twofold increase in soluble intracellular αSyn • Significantly increased monomeric αSyn in inferior temporal cortex of AD cases versus MCI and NCs	• Larson et al. 2012 [69]
• Brain tissue	• *N*=542 - AD-LBV (*n*=308) - DLB (*n*=13) - PD (*n*=141) - PD with AD-pathology (*n*=80)	• AD-LBV patients had distinct and prominent LRP in the amygdala, limbic and olfactory systems, with little/no brainstem LRP	• Toledo et al. 2016 [70]
• Clinicopathological correlations	• *N*=59 - AD (*n*=19) - DLB (*n*=18) - AD+DLB (*n*=22)	• 50% of AD+DLB, 94% of DLB and 16% of AD cases had complex visual hallucinations - thus, within the context of AD-type dementia, visual hallucinations may indicate possible LRP	• Thomas et al. 2018 [71]

*AD* Alzheimer’s disease, *αSyn* α-synuclein, *NC* non-demented control, *AD-LBV* Alzheimer’s disease Lewy body variant, *LBs* Lewy bodies, *LN* Lewy neurites, *DLB* dementia with Lewy bodies, *LRP* Lewy related pathology, *Aβ* amyloid-β, *SNc* substantia nigra, *APOEε4* apolipoprotein ε4 allele, *NFTs* neurofibrillary tangles, *ADAD* autosomal dominant Alzheimer’s disease, *PSEN1* presenilin 1 allele, *PSEN2* presenilin 2 allele, *APP* amyloid precursor protein allele, FTD frontotemporal dementia, *VsD* vascular dementia, *MCI* mild cognitive impairment, *PD* Parkinson’s disease, *AD+DLB* co-diagnosis of Alzheimer’s disease and dementia with Lewy bodies

Interestingly, the co-occurrence of LRP in AD is frequently associated with the immunohistochemical co-localization of αSyn and tau pathology [22, 24, 61, 63], and to a lesser extent αSyn and Aβ pathology [62, 65]. Co-immunoprecipitation experiments have shown that αSyn monomers, dimers, trimers and pentamers bind to monomeric Aβ in post-mortem samples from AD and DLB brains [67]. Importantly, two reports from Larson, Lesné and co-workers demonstrated significantly increased levels of intracellular soluble αSyn monomers and oligomers in the inferior temporal lobe of AD brains without any detectable LRP [69, 73]. Specifically, when compared to brain samples from cognitively healthy controls, post-mortem AD brains exhibited increased 17-, 28-, 35- and 56-kDa, but not 72-kDa αSyn species, which correlated to AD-related cognitive decline and reduced synapsin expression within the temporal lobe. Thus, an increased presence of potentially toxic forms of soluble intracellular αSyn, direct interactions between oligomeric αSyn and monomeric Aβ, and the frequent co-localization of αSyn with NFTs and Aβ plaques in brain regions susceptible to AD neurodegeneration may explain the more severe clinical symptoms often found in AD patients with co-occurring LRP. More importantly, the results provided by Larson and colleagues [69, 73] suggest that soluble αSyn and the local concentrations thereof may play an important role in AD regardless of LRP. It is also pertinent to note that LRP [74] and AD-type pathology [75] have been documented in cognitively healthy individuals upon autopsy. For instance, 24% of the autopsied normal control cases from the University of Kentucky Alzheimer’s Disease Center exhibited LRP [74]. This has led to the speculation that these subjects harboring asymptomatic pathology are in a presymptomatic disease stage [74], whilst others argue that insoluble aggregates are inherently inert and/or protective [76]. Yet others have postulated that protein toxicity is context-dependent [77].

## Cerebrospinal fluid α-synuclein levels in synucleinopathies versus AD

Whether accumulations of LRP or altered local concentrations of αSyn in the brain parenchyma can be monitored in CSF, similar to the situation with Aβ and tau [49, 78], remains to be established. Accumulating evidence from our own laboratory and those of others have shown that patients with synucleinopathies, mainly Parkinson’s disease (PD) and DLB, frequently exhibit reduced levels of CSF αSyn [19, 79–81], for an overview of the literature please see Table 2. Interestingly, reduced CSF αSyn levels do not appear to be associated with clinical or imaging measures of PD severity [95]. Although a substantial amount of results now propose a synucleinopathy-specific reduction of CSF αSyn, consensus on a CSF profile of reduced αSyn in synucleinopathy patients is still lacking mainly due to the high variability between the various αSyn quantification assays, differences in the species of αSyn being quantified and inter-laboratory variability [96]. Nevertheless, several studies including our own have shown that lower CSF αSyn could distinguish PD from AD patients [81, 97]. In contrast to the reported lower CSF αSyn levels in patients with PD and DLB, numerous studies have documented unaltered or slightly increased CSF αSyn levels in patients with MCI and AD [27, 80–82, 86, 90, 91, 93, 94]. Higher CSF αSyn levels were also shown to be positively associated with disease progression from sporadic MCI to AD [27, 92] and negatively correlated to cognitive function (MMSE scores)[27, 86, 93]. Our own study in addition exhibited an effect of the *APOE*ε4 variant in MCI patients that progressed to fulfill the diagnostic criteria for AD over a 24 month period. In these patients we documented a dose-response relationship between the *APOE*ε4 allele and CSF αSyn levels where *APOE*ε4 homozygous carriers exhibited the highest CSF αSyn concentrations [26]. Furthermore, in a cross-sectional sample of participants carrying ADAD mutations from the Dominantly Inherited Alzheimer Network (DIAN) we found that CSF αSyn levels were significantly correlated to the estimated years from disease onset [27]. We also found that specifically in asymptomatic (CDR<0.5) DIAN participants who carried ADAD mutations and who also were *APOE*ε4 positive, higher CSF αSyn levels significantly correlated to PiB-PET Aβ plaque burden in several of the ‘early Aβ-accumulating brain areas’. Interestingly, the direction of this relationship between CSF αSyn levels and brain Aβ plaque burden was positive in DIAN participants that were asymptomatic, however, the direction changed to negative when symptoms began to surface (CDR>0.5). Although we found significant associations between CSF αSyn levels and PiB-PET Aβ burden, we, rather unexpectedly, failed to record any significant correlations between CSF αSyn and Aβ_1-42_ [27]. A relationship between brain Aβ load and CSF αSyn levels was previously proposed by Vergallo and co-authors who examined cognitively normal individuals with subjective memory complaints stratified by AD biomarkers [26]. The same study further confirmed the by us and others previously reported strong correlation between CSF levels of αSyn and both t-tau and p-tau [19, 81, 98]. The biological relevance of the consistently reported correlation between αSyn and tau in CSF is not known. However, both αSyn and tau appear to be secreted by similar exosome-mediated release mechanisms [99, 100] and both proteins can also be endocytosed via the heparin sulfate proteoglycans (HSPGs) pathway [101]. Hence, the extracellular levels of αSyn and tau may be regulated by similar mechanisms reflected in a strong consistent correlation between the CSF concentrations of these proteins in both control subjects and AD patients. Together these results suggest an association between biological processes reflected in altered CSF αSyn levels and the asymptomatic development of AD pathology, which in turn may be under the influence of *APOE*ε4.

**Table 2:** Clinical evidence supporting a role for α-synuclein in AD pathophysiology

Type of Analysis	Cohort	Findings	Citation (Year)
• CSF	*N*=80 - AD (*n*=13) - DLB (*n*=38) - PD (*n*=8) - CJD (*n*=8) - NC (*n*=13)	• CSF αSyn levels: - higher in AD versus synucleinopathies - no difference in AD versus NC	• Mollenhauer et al. 2008 [82]
• CSF	*N*=151 - AD (*n*=66) - DLB (*n*=15) - PD (*n*=15) - NC (*n*=55)	• CSF αSyn levels lower in AD patients versus NCs • In AD patients, lowest CSF αSyn associated with MMSE<20	• Ohrfelt et al. 2009 [83]
• CSF	• *N*=86 - AD (*n*=31) - DLB (*n*=34) - other dementias (*n*=21)	• CSF αSyn levels: - Lower in DLB versus AD and other dementia patients - No difference in AD versus DLB or other dementia patients	• Kasuga et al. 2010 [84]
• CSF	• *N*=150 - AD (*n*=63) - DLB (*n*=35) - PD (*n*=18) - subjective memory complaints (*n*=34)	• CSF αSyn levels did not differ between patient groups • Lower CSF αSyn in DLB patients marginally associated with reduced cognitive performance	• Reesink et al. 2010 [85]
• CSF	• *N*=191 - AD (*n*=46) - DLB (*n*=33) - PDD (*n*=22) - PD (*n*=38) - NC (*n*=52)	• CSF αSyn levels higher in AD patients compared NCs • CSF αSyn levels lower in synucleinopathies versus AD and NCs • CSF αSyn levels were positively correlated with p-tau in all groups, and t-tau in all groups except PD/PDD	• Wennström et al. 2013 [81]
• CSF	• *N*=117 - AD (*n*=36) - MCI (*n*=65) - NC (*n*=16)	• Increased CSF αSyn in AD and MCI versus NC • Increased CSF αSyn levels correlated to decreased MMSE score in combined AD and MCI patient group	• Korff et al. 2013 [86]
• CSF	• *N*=68 - AD (*n*=18) - DLB (*n*=16) - NC (*n*=22)	• Increased CSF αSyn levels in DLB versus AD and NC	• Kapaki et al. 2013 [87]
• CSF	• *N*=389 (AD cohort) - AD (*n*=92) - MCI (*n*=187) - NC (*n*=110) • *N*=102 (PD cohort) - PD (*n*=63) - NC (*n*=39)	• Increasing CSF αSyn levels negatively correlated to p-tau levels in AD cohort (AD and MCI patients combined), but not in the PD cohort. • CSF αSyn and t-tau levels positively correlated in both cohorts and cohort patient groups	• Toledo et al. 2013 [88]
• CSF	• *N*=247 - AD (*n*=48) - DLB (*n*=71) - PDD (*n*=30) - NC (*n*=98)	• CSF αSyn oligomer levels significantly higher in DLB and PDD versus AD patients	• Hansson et al. 2014 [89]
• CSF	• *N*=262 - probable AD (*n*=94) - AD (*n*=34) - probable DLB (*n*=30) - DLB (*n*=15) - probable PDD (*n*=23) - probable PD (*n*=30) - probable MSA (*n*=7) - MSA (*n*=1), (ante-mortem) NC (*n*=29)	• CSF αSyn levels higher in AD patients versus synucleinopathy patients and HCs • CSF αSyn levels correlated to CSF t-tau and p-tau in autopsy-confirmed AD patients • CSF αSyn combined with CSF t-tau and p-tau may be able to differentially diagnose AD from DLB patients	• Slaets et al. 2014 [90]
• Meta-analysis	• *N*=10 research articles	• CSF αSyn levels were significantly higher in AD versus DLB, PD and MSA patients	• Wang et al. 2015 [91]
• CSF	• *N*=99 - AD (*n*=26), - MCI converting to AD (*n*=25) MCI-stable (*n*=23) - NC (*n*=25)	• In MCI patients who converted to AD within two years CSF αSyn levels were substantially higher and associated with an aggressive symptom onset	• Berge et al. 2016 [92]
• CSF	• *N*=293 - AD (*n*=225) - NC with other neurological diseases (*n*=68)	• In AD patients versus controls CSF αSyn: - positively correlated to CSF t-tau and p-tau - positively correlated to decreasing MMSE score	• Majbour et al. 2017 [93]
• Brain tissue	*N*=84 • AD (*n*=24), MCI (*n*=34), NC (*n*=26)	• AD brain samples without LRP had increased intracellular monomeric and oligomeric αSyn with weights varying from 17- to 54-kDa	• Larson et al. 2018 [73]
• CSF	• *N*=367 Clinical cohort - AD (*n*=114), MCI (*n*=63), DLB (*n*=10), PD (*n*=32), bvFTD (*n*=48), CBS (*n*=8), PSP (*n*=9), ALS (*n*=35), NC (*n*=48) • *N*=71 Autopsy cohort - AD (*n*=29), PD (*n*=3), PD with AD pathology (*n*=4), AD with LRP (*n*=12), LRP-TDP (*n*=2), FTLD (*n*=12), FTLD-AD (*n*=4), ALS (*n*=5)	• CSF αSyn levels were higher in clinical AD and autopsy-confirmed AD cohorts • The addition of CSF αSyn to the standard CSF AD biomarker panel improved the differential diagnosis of AD versus synucleinopathies (PD, PDD, DLB) and FTD	• Shi et al. 2018 [94]
• CSF • Neuroimaging	• *N*=36 subjective memory complaint patients - PET-Aβ(+) (*n*=8) - PET-Aβ(-) (*n*=28)	• Increased CSF αSyn associated with increased brain Aβ deposition • CSF αSyn positively correlated to CSF t-tau and p-tau	• Vergallo et al. 2018 [26]
• CSF • Neuroimaging	• *N*=136 AD cohort - AD (*n*=27) - MCI converting to AD (*n*=27) - MCI remaining MCI (*n*=27) - NC (*n*=57) • *N*=142 ADAD cohort - *APP* (*n*=24) - *PSEN1* (*n*=50) - *PSEN2* (*n*=18) - related non-ADAD mutation carriers (*n*=50)	• In the sporadic AD cohort: - at baseline CSF αSyn levels were increased in MCI patients who converted to AD after two years (MCI-AD) - MCI-AD patients also exhibited a positive dose-dependent association between CSF αSyn levels and the *APOEε4* allele • CSF αSyn levels were positively associated with t-tau and p-tau in all investigated groups • In the ADAD cohort: - asymptomatic ADAD mutation carriers, and *APOEε4* positive ADAD mutation carriers had significant positive association between increased CSF αSyn levels and brain Aβ deposition in regions know to exhibit early Aβ deposition - CSF αSyn levels were higher in symptomatic ADAD mutation carriers versus asymptomatic ADAD mutation carriers - CSF αSyn levels positively correlated with estimated years from symptom onset in ADAD mutation carriers • CSF αSyn levels were positively correlated with t-tau in non-mutation and ADAD mutation carriers, and with p-tau in non-mutation carriers and *PSEN1* mutation carriers only (not *APP* or *PSEN2* mutation carriers)	• Twohig et al. 2018 [27]

*AD* Alzheimer’s disease, *DLB* dementia with Lewy bodies, *PD* Parkinson’s disease, *CJD* Creutzfeldt-Jakob Disease, *NC* non-demented control, *CSF* cerebrospinal fluid, *αSyn* α-synuclein, *MMSE* Mini Mental State Examination, *MSA* multiple system atrophy, *PDD* Parkinson’s disease with dementia, *MCI* mild cognitive impairment, *p-tau* phosphorylated tau, *t-tau* total tau, *bvFTD* behavioral variant frontotemporal dementia, *CBS* corticobasal syndrome, *PSP* progressive supranuclear palsy, *ALS* amyotrophic lateral sclerosis, *FTD* frontotemporal dementia, *LRP-TDP* Lewy body related pathology with transactive response DNA-binding protein 43 pathology, *LRP-AD* Lewy body related pathology with Alzheimer’s disease pathology, *FTLD* frontotemporal lobar degeneration, *FTLD-AD* frontotemporal lobar degeneration with Alzheimer’s disease pathology, *LB* Lewy body, *LRP* Lewy related pathology, *VaD* vasculature dementia, *ADAD* autosomal dominant Alzheimer’s disease*, PSEN1* presenilin 1 allele , *PSEN2* presenilin 2 allele, *APP* amyloid precursor protein allele, Aβ amyloid-β, *PET* positron emission tomography, *APOEε4* apolipoprotein ε4 allele

## α-synuclein structure

**Fig. 1:**
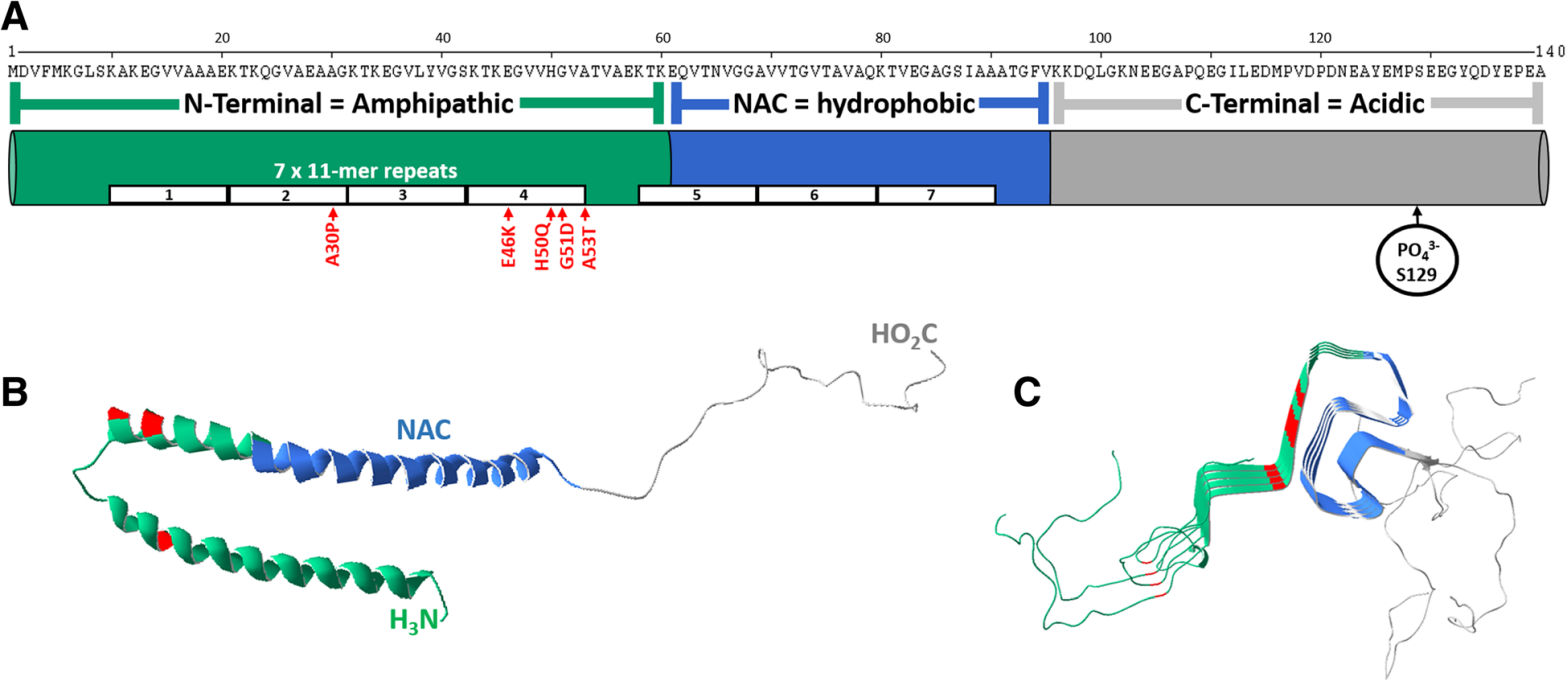
The structures of αSyn. **a** Primary structure of αSyn. N-terminal residues are green, the non-amyloid component (NAC) residues are blue, C-terminal residues are grey and disease associated point mutations are red. **b** Molecular model of a crystal structure of micelle bound human αSyn, PDB 1XQ8[103]. Color scheme is same as in **a**. **c** Molecular model of a cryo-electron microscopy structure of four human αSyn proteins (residues 1-121) in a fibril, PDB 6H6B[104]. Color scheme is same as in **a**. Molecular models created using Deep View Swiss PDB Viewer [105]

α-synuclein is a small 140-residue (14-kDa, p*K*_a_= 4.7) protein translated from 5 exons of the *SNCA* gene located on the long arm of chromosome four at position 21 (4q21.3-q22) [102]. The primary structure of αSyn (Fig. 1a) is subdivided into three regions: an amphipathic N-terminal (amino acids (a.a.) 1-60), a hydrophobic core region known as the NAC (a.a. 61-95), and an unstructured acidic C-terminal (a.a. 96-140). Although the overall secondary structure of αSyn has yet to be determined there is a consensus that the N-terminal and NAC can together fold into α-helices (Fig. 1b). Multiple in vitro experiments have shown that the N-terminal and NAC of αSyn can form a single α-helix (a.a. 3-92) when bound to membranes of low curvature [106–109] or two amphipathic anti-parallel α-helices (a.a. 3-37 and a.a. 45-92) when bound to lipid membranes of high curvature [103, 110, 111] (Fig. 1b). Within the N-terminal and NAC are seven highly conserved imperfect 11-residue repeats based on the XKTKEGVXXXX motif which form the structural backbone of the α-helices [112] and may also stabilize the native conformations of αSyn [113, 114] (Fig. 1a). The NAC appears to be essential in the aggregation of αSyn due to its ability to adopt β-sheet structures (Fig. 1c) [115] and has also been shown elicit seeding-competent amyloids [116, 117]. Introducing hydrophilic point mutations within the NAC significantly reduces the oligomerization and fibrillation potential of αSyn, further suggesting that the NAC plays a central role in the aggregation of αSyn [116]. These 11-residue repeats are also found in other lipophilic proteins such as apolipoproteins which also form α-helices that interact with membranes, however the a.a. sequence is unique to the synucleins alone [118]. A noteworthy report found that the loss of two repeat motifs led to the formation of β-sheet enriched oligomers and amyloid fibrils, while adding two additional repeats inhibited the formation of both β-sheet enriched oligomers and amyloid fibrils (Fig. 1c). These findings thus highlight the structural and physiological importance of the highly conserved features of αSyn [119]. Importantly, all known autosomal dominant point mutations that cause PD reside within these repeat motifs, specifically A30P[120], E46K[121], A53T[122], G51D[123], and H50Q[124] (Fig. 1a). The location and severity of these pathogenic mutations shows the important relationship between the structure and function of αSyn (Fig. 1b-c).

The C-terminal of αSyn (a.a. 96-140) is inherently acidic and remains disordered even as the N-terminal adopts secondary structures [125]. There is considerable debate regarding the inherent role of the C-terminal [126] with studies suggesting that it: i) interacts with the N-terminal or NAC to stabilize the native unfolded structure thus preventing fibrillation [127], ii) interacts with the N-terminal or NAC to form compacted monomeric structures which resist the propensity to aggregate at low temperatures [128, 129], iii) binds to calmodulin [130, 131], iv) binds to Fe^3+^ during αSyn-catalyzed Fe^3+^→ Fe^2+^ reduction [132], or iv) binds to both calcium and synaptic vesicles thereby influencing synaptic vesicle clustering, αSyn aggregation kinetics and/or dopamine-induced cytotoxicity [133].

Many reports have confirmed the widely held assumption that αSyn primarily exists as an unfolded protein in dynamic equilibrium with smaller pools of membrane-bound αSyn, however, others have advanced the theory that αSyn exists in pools of soluble helical multimers [134]. The role and nature of αSyn multimers are unclear. Burré and colleagues reported that αSyn multimers assemble when bound to vesicles engaged in SNARE dependent exocytosis [135], while Dettmer and co-authors instead postulated that membrane-free αSyn multimers, specifically αSyn tetramers, form the largest steady-state pool of intracellular αSyn [134]. The latter group has also reported that in human brain lysates, human neuronal cultures, mouse models, and in mouse/human/goat cell lines expressing familial PD αSyn mutations (A53T or E46K), a decrease in the tetramer:monomer ratio correlated to increased neurotoxicity. This led the authors to conclude that αSyn tetramers and low molecular weight multimers disassemble into less-stable higher-energy monomers in order to bind to membranes, however, when monomers are in excess they are also potentially neurotoxic [136–140].

Thus, αSyn appears to exist in a dynamic physical equilibrium fluctuating between potentially numerous metastable homogenous or heterogeneous secondary, tertiary and quaternary structures involving numerous binding partners and physiological processes. To improve our understanding of whether specific species of αSyn lose or gain functions that may promote or intermingle with AD pathophysiological processes more detailed studies of αSyn in the context of AD are needed.

## α-synuclein expression and function

**Fig. 2:**
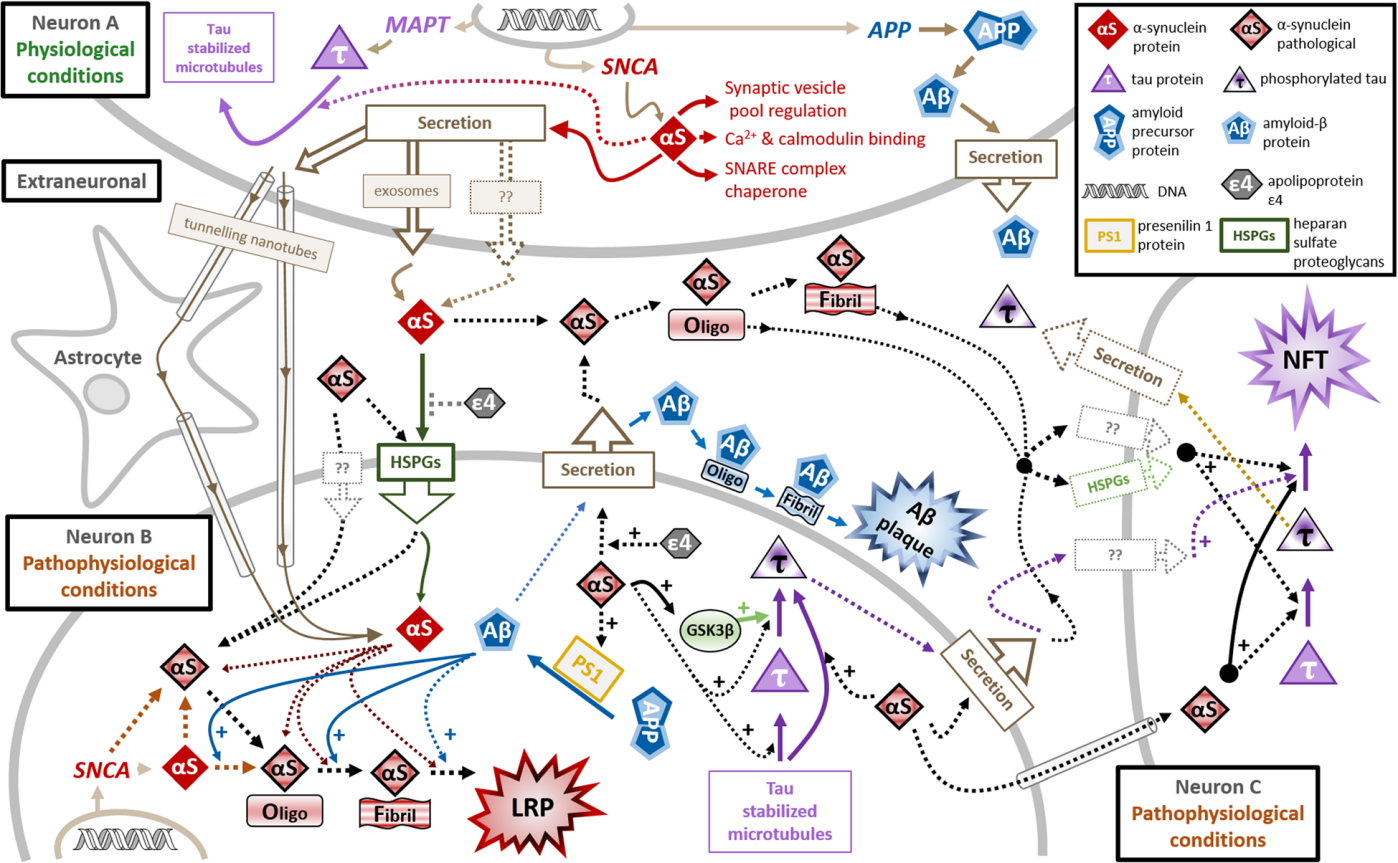
Known (solid lines) and hypothetical (dotted lines) mechanisms shuffling αSyn between the intra-and extracellular compartments with implications for the development of AD pathological lesions. The top left-hand side and middle of the figure depicts physiological processes taking place in the healthy neuron A, the middle of the figure (underneath the healthy neuron A) is the extra-neuronal space with an astrocyte. The bottom half and right-hand side of the figure depicts two independent neurons in pathophysiological conditions, neurons B and C respectively

Although abundantly expressed in the kidneys and blood cells, αSyn is predominantly expressed within the presynaptic terminals of neurons [141–143] and can also be found in the neuronal nucleus where it appears to co-localize with the nuclear membrane [144]. Expression of αSyn can mainly be found in the cerebral cortex, cerebellum, striatum, thalamus, hippocampus and olfactory bulb [141, 145]. The factors regulating the transcription of αSyn remain largely unknown, however, *SNCA* has shown an affinity for zinc-finger motifs and responds accordingly to the transcription factors GATA-binding factor 2 (GATA2) [146] and zinc finger and SCAN domain-containing protein 21 (ZSCAN21, [147]. Similar to tau, it has been suggested that αSyn is secreted within exosomes in a calcium-dependent manner [100]. It was further proposed that αSyn can be taken up by cells via HSPGs similar to what has been reported for tau and Aβ [101](Fig. 2). Interestingly, APOE was shown to reduce cellular uptake of Aβ by competing for HSPGs [148] and specifically the APOE4 isoform limited the cellular uptake of αSyn in oligodendrocytes [149]. Several mechanisms have been reported to orchestrate the degradation of αSyn including autophagy and catabolism mediated by the ubiquitin-proteasome system [150]. Also, extracellular proteases like neurosin, also referred to as kallikrein 6, have been shown to degrade αSyn [151, 152]. Notably, the lentivirus-facilitated expression of neurosin in a mouse model of Lewy body disease enhanced clearance of αSyn and reduced αSyn pathology [153]. Hence an interplay between various molecules including APOE may determine the distribution of αSyn between the extracellular and intracellular pools. The balance between these two pools in turn may determine the physiological roles of αSyn in both compartments.

Although the native role of αSyn is unclear it has been strongly linked to various pre-synaptic processes. Initially αSyn was suggested to play a role in learning and plasticity after George and colleagues reported upregulation of αSyn RNA in the zebra finch song control circuit during a period of song-acquisition-induced synaptic re-arrangement [154].

The first in vivo forays into Syn mouse models showed that αSyn knock-out (KO)[155, 156], β-Syn KO [157], α/β-Syn double-KO [157], and γ-Syn KO [158] animals were all viable, fertile, displayed normal brain development, morphology, synaptic ultrastructure, they had no behavioral abnormalities, and lacked any distinguishable phenotype [155–158]. However, αSyn-KO, and α/β-Syn double-KO mice exhibited reduced brain dopamine levels compared to wild-type (wt) animals and markedly different responses to electrophysiological interrogation were documented in three different studies. One study reported no differences [157], another study found disinhibition of synaptic depression following paired-pulses [155], and yet another noted prolonged recovery times after a train of stimuli pointing to αSyn having a roll in vesicle trafficking or refilling [148]. Studies using rat primary dopaminergic cell models showed that overexpression of αSyn via viral injection into the substantia nigra of mature animals reduced dopamine release and reuptake by 80-90% [159]. Therefore, one could speculate that αSyn and β-Syn may functionally compensate for one another, and that αSyn may be involved in the long-term synaptic regulation and or maintenance of dopaminergic pathways. The α/β/γ-Syn triple-KO animals were viable but had lower survival rates and age-dependent neuronal dysfunction in the hippocampus and retina [160]. This was not due to a loss of synapses, but rather due to changes in synaptic protein composition and alterations of axonal and synaptic architecture, thereby augmenting the fidelity of the signal. The electrophysiological differences between synuclein null and wt animals were shown to start at a young age but could be rescued by both human and murine αSyn expression [160], again indicating that αSyn has a presynaptic role. Mice harboring a deletion of the presynaptic SNARE complex chaperone cysteine string protein-α (CSPα) had significant neurodegeneration, shortened survival (<4 months), reduced levels SNAP-25, and hindered SNARE-complex assembly [161]. Remarkably, transgenic expression of αSyn completely rescued the neurodegeneration in CSPα^-/-^ animals, while loss of endogenous αSyn exacerbated the neurodegeneration [161], indicating that αSyn has a compensatory, neuroprotective role within the presynaptic SNARE machinery.

Further support for the notion that αSyn has an important role within the presynaptic machinery, specifically in regards to vesicle dynamics, also came from rat primary cell culture studies where αSyn was shown to exclusively co-localize with the transmembrane synaptic vesicle protein synaptophysin [162]. Ultrastructural analysis further revealed that knock-down of αSyn resulted in a significant reduction in the distal/reserve pool of synaptic vesicles [162]. Single molecule fluorescence studies recently have also determined that 70-αSyn units bind per presynaptic vesicle, which is very similar to the ratio of synaptobrevins per vesicle, further indicating αSyn’s involvement in presynaptic vesicle trafficking and/or remodeling. A more detailed investigation regarding the potential interactions between αSyn and synaptic vesicles was conducted using transgenic mice overexpressing human αSyn tagged with GFP, and found that the transgenic neuronal cultures had augmented synaptic architecture, reduced pre-synaptic protein levels and altered release kinetics [163]. Specifically, the primary transgenic neuronal cultures exhibited a reduction in the density of pre-synaptic vesicles and a more heterogeneous distribution of synaptic vesicle sizes with more unusually large (>50nM) vesicles. The same study further showed reduced levels of VAMP2, piccolo, synapsin-1 and amphiphysin paralleled by a reduced frequency of spontaneous neuronal activity [163]. Importantly, these changes were also correlated to increasing levels of proteinase K-resistant αSyn and phosphorylated αSyn (pS129), thereby providing a potential link between synaptic dysfunction and pathological αSyn alterations. Using the same model system it was later confirmed that overexpression of αSyn lead to a specific reduction in in size of the recycling pool of synaptic vesicles and hindered intersynaptic vesicle trafficking compared to wt cultures [164]. Moreover, αSyn-KO cultures showed the opposite effect, as they had a larger recycling pool and increased intersynaptic trafficking of synaptic vesicles. Interestingly, further experiments using transcriptionally encoded human αSyn dimers overexpressed in dissociated αSyn-KO hippocampal neurons resulted in an increased clustering of synaptic vesicles, retarded vesicle motility and augmented recycling kinetics compared to wt neurons [165]. The increased ability of αSyn multimers to cluster synaptic vesicles was significantly reduced by the introduction of seven hydrophilic lysine residues which were thought to disrupt the membrane associated αSyn α-helix, but somewhat surprisingly, there was no difference in synaptic vesicle binding compared to wt αSyn [165]. These findings were further verified and expanded upon by Nemani et al. using murine hippocampal neurons overexpressing human αSyn. They found that compared to wt neurons, αSyn overexpression reduced the size of the reserve- and ready releasable pools of synaptic vesicles, inhibited post-endocytosis vesicle clustering and attenuated SNARE mediated exocytosis [166].

The dynamics of the presynaptic exocytotic/endocytotic fusion pore are also potentially mediated in part by αSyn. Logan et al. showed that overexpressing human αSyn in WT mice neurons retarded the closure of the fusion pore, reduced the number of exocytotic events, but also increased the kinetics of exocytosis which may be the neurons attempt to compensate for the loss in exocytosis frequency [167]. Moreover, when the same authors used wt mice chromaffin cells expressing the A35T αSyn mutation dilation, rather than closure, of the fusion pore was impaired [167, 168].

We find it rather odd that a protein so highly conserved, synaptically enriched, and potentially toxic is at the same time non-essential, and non-specific in its function. We speculate that the transient nature of αSyn’s inherent structures and the large variation in murine models, cell lines and the use of various non-standardized peptides and proteins contribute to the previously reported variation in outcome [169]. To improve our understanding of αSyn during normal and pathological conditions such as in AD, concerted efforts using both in vitro and in vivo models, preferably in a translational manner, and techniques that readily can capture the various molecular species of αSyn are highly warranted.

## Experimental evidence supporting a role for α-synuclein in AD pathophysiology

To improve the understanding of potential molecular pathways through which αSyn may be connected to the pathophysiology of AD and neurodegeneration at large, various studies have employed transgenic AD mouse lines in which αSyn was either manipulated, reduced or eliminated. The results of these various studies are however not fully consistent and consensus-building conclusions remain to be drawn. We speculate that at least some of these inconsistencies depend on inter-model variabilities. For example, Spencer and colleagues reported that APP Tg/α-syn KO animals exhibited reduced cholinergic and hippocampal neurodegeneration, less behavioral deficits and increased expression of Rab proteins [170]. A similar APP/αSyn-KO mouse model in the hands of Khan and co-workers exhibited enhanced memory performance but significantly aggravated Aβ plaque pathology. The same authors also showed that elimination of αSyn prevented premature death in the APP mice paralleled by a remarkable rescue of behavioral parameters and prevention of pathological neuronal cell cycle re-entry (CCR). The APP/αSyn-KO mice further exhibited reduced levels of extracellular Aβ oligomers and less αSyn oligomers. In addition, Khan et al. also overexpressed human wt-αSyn in mutant APP mice (APP/αSyn) and found that the behavioral and pathological phenotypes had reversed compared to the APP/αSyn-KO mice. Although the APP/αSyn mice had a significantly reduced Aβ plaque burden, they had exacerbated behavioral deficits, more deleterious neuronal cell cycle re-entry, and increased levels of Aβ oligomers, αSyn oligomers and tau pathology [171]. The expression of APP in the two APP/αSyn-KO mouse models used by Spenser and Khan respectively, was driven by either the mouse Thy1 or the PGDFβ promoter [170, 171]. We speculate that the variability in the reported Aβ pathology between these mouse models may be directly linked to the regulation of APP expression in these mice.

Another remarkable study showed that cortical shRNA-induced reduction of *SNCA* expression in rats with complete T10-T11 spinal cord transections, resulted in a significant increase in neuronal survival, decreased apoptosis, and stimulated motor and sensory recovery in the hindquarters. In contrast upregulation of SNCA expression exacerbated neuronal death and impeded recovery [172]. The neuroprotective mechanism conferred by down-regulating or KO of αSyn is unknown but may be due to an enhanced ability to sequester toxins in vesicles [173], or through reducing potentially pathologic post-translational modifications of αSyn [174]. Further, the reduction of αSyn in αSyn-KO/KD models could regulate important hippocampal glutamatergic mGluR5 and NMDA receptors [175], and has the potential to regulate various members of the Rab GTPase family which are key intracellular membrane trafficking proteins [175, 176].

Identification of potential mechanisms specifically linking αSyn to AD pathophysiology, in addition to the prominent roles αSyn appears to play in orchestrating synucleinopathies like PD, DLB and MSA, would offer crucial insights into disease-specific pathogenic processes and hopefully allow for the development for more potent interventions. A multitude of studies have already shown molecular interactions between αSyn and tau which may have implications for various neurodegenerative diseases [177]. For example, the C-terminal of αSyn can directly interact with the microtubule binding domain of tau [178], and αSyn seeds have been shown to induce intracellular aggregation of tau [179]. Interactions between tau and αSyn could potentially also be mediated by the serine/threonine kinase glycogen synthase kinase 3-β (GSK3β)[180]. The GSK3β participates in multiple cellular signaling pathways related to development, proliferation, viability, immunity and apoptosis. The GSK3β can orchestrate pathological tau-phosphorylation at over two-dozen disease-associated sites [181–184]. Other reports have suggested that GSK3β activity could be increased by Aβ, inhibited acetylcholine synthesis [185], and could trigger apoptosis [183]. Hence, the active role GSK3β plays in AD pathophysiology appears to be established. Importantly, several lines of evidence propose an association between GSK3β and αSyn. Kozikowski and co-authors used human neuroblastoma SH-SY5Y cells stably expressing αSyn to screen novel GSK3β inhibitors, and found that the selective inhibition of GSK3β not only prevented tau phosphorylation but also reduced αSyn protein levels [186]. Later reports from different groups confirmed the findings of Kozikowski and colleagues and further demonstrated that αSyn can regulate the activity, but not the expression of GSK3β[187]. Interestingly, the αSyn-NAC region can directly mediate the activity of GSK3β by inducing GSK3β autophosphorylation leading to GSK3β-mediated tau-hyperphosphorylation [180]. However, this was not αSyn specific as β-synuclein was also shown to mediate the same signaling cascade [180].

Whether extracellular levels of αSyn would be of relevance to the activity of GSK3β and its’ effects on tau protein was recently investigated in vitro by Gassowska and colleagues using the PC12 cell line. They found that exogenously added αSyn increased the phosphorylation of tau at the Ser-396 site which could be prevented by the GSK3β inhibitor SB216763[188]. The same study further showed that αSyn both increased the activity of GSK3β and the protein levels thereof [188]. Whether alterations in both extracellular versus intracellular levels of αSyn specifically could promote the actions of GSK3β is an important question, highlighted by the previously described inverse alterations in CSF αSyn levels in patients with synucleinopathies compared to AD patients. Importantly, the processes governing the distribution of αSyn between the extracellular and intracellular pools may be a crucial factor in pathological downstream processes such as tau phosphorylation. As mentioned previously, αSyn can be degraded extracellularly by proteases like neurosin [151, 152] but also intracellularly by lysosomes through the chaperone-mediated autophagy pathway [189]. The latter was also confirmed to occur in vivo during normal and pathological conditions [190]. Similar to tau, αSyn can be endocytosed via the HSPG pathway [101]. This observation offers an opportunity to better understand what biological processes may underlie the increased disease risk of the *APOE*ε4 allele for both AD and DLB [16], as APOE may be involved in the distribution of αSyn between the intracellular and extracellular space. For example, APOE was shown to compete with Aβ for cellular uptake in vitro via the HSPG pathway thereby limiting Aβ uptake [148], and APOE4 can also specifically reduce αSyn uptake in primary human oligodendrocytes [149]. With APOE as an important lipid-carrier in both the CNS and the periphery it is tempting to speculate that the *APOE* allele-associated alterations in lipid profiles may be linked to various Aβ, tau and αSyn pathological processes due to their ability to interact with free lipids and membranes [191, 192]. Interestingly, an age-dependent reduction in glucocerebrosidase (GCase) activity in mice was found to be associated with lipid-dependent changes in neuronal vesicular membrane compartments which accumulated lipid-stabilized αSyn and phosphorylated tau [193]. The authors specifically commented on the loss of GCase activity which under normal conditions results in the conversion of glucosylceramide into ceramide and glucose [194], and its association with lipid homeostasis in PD and DLB. They further reported a significant correlation between GCase activity and αSyn aggregation in the human PD brain which prompted them to propose that age-associated lipid changes may be related to αSyn and tau pathology [193]. We speculate that an age-related lipid dyshomeostasis could be further aggravated by *APOE*ε4 which down-stream could translate into altered intra- versus extracellular levels of αSyn. Consequently we could hypothesize that in prodromal AD patients [27] the link between *APOE*ε4 and higher CSF αSyn levels may be attributed to higher extracellular levels of αSyn due to a shift in the αSyn intra- versus extracellular pools possibly driven by either lipid-related changes or hampered cellular αSyn uptake in the presence of the APOE4 isoform. Further evidence linking APOE to αSyn were previously exhibited from transgenic mouse models expressing mutant human A30P αSyn (αSyn-A30P)[195]. The αSyn-A30P transgenic mice exhibited behavioral dysfunction, spinal cord astrocytosis, spinal cord motor neuron degeneration, increased levels of both insoluble αSyn and Aβ, and a substantial (4-fold) increase in APOE [195]. This strong effect that mutant αSyn had on APOE led the authors to cross the A30P transgenic mice with wt ApoE-KO mice (A30P/ApoE-KO). The resulting A30P-ApoE-KO mice still had neurodegeneration, but had increased survival rates, delayed onset of behavioral symptoms, reduced neurodegeneration, and significant reductions of Aβ aggregates [195]. Remarkably, knocking out *APOE* also increased the levels of monomeric αSyn, while reducing levels of oligomers and other larger αSyn aggregates.

Hence, *APOE*ε4, which has been implicated in AD pathophysiology and pathogenesis by processes mainly linked to Aβ but also associated with Aβ-independent pathways [15], may be the missing link connecting Aβ to tau via actions of αSyn. This notion is supported by evidence showing an effect of αSyn on Aβ itself. For example, in 2006 Mandal and colleagues used nuclear magnetic resonance spectroscopy to investigate the interactions between membrane bound αSyn with Aβ_1-40_ or Aβ_1-42_ and found that both forms of Aβ interacted with bound αSyn [196]. Notably, αSyn enhanced the toxic oligomerization of Aβ_1-42_ leading to oligomeric Aβ_1-42_ mediated cleavage of αSyn and liberation of the NAC, therefore providing a plausible mechanism explaining why the NAC co-aggregates in Aβ plaques in vivo (Fig. 2). In addition, in vitro and in vivo models have shown that αSyn can interact and catalyze the oligomerization of tau [197], and form neurotoxic cation pores with Aβ [67].

Further evidence of a link between αSyn and Aβ pathology was offered from studies using mice expressing wt human αSyn (hαSyn) and human APP (hAPP) (hαSyn/hAPP). The hαSyn/hAPP model recapitulated many of the clinical symptoms and pathology of AD-LBV. Compared to animals expressing only hαSyn or hAPP, the hαSyn/hAPP animals had marked cholinergic neuron loss, intraneuronal LRP, florid Aβ plaques, exhibited marked motor dysfunction and had significant learning and memory deficits [198]. Animals expressing all three AD pathologies (Aβ, tau and αSyn) were developed by crossing 3xTg-AD mice (APP^KM670/671NL^_,_ tau^P301L^, PS1^M146V^) with αSyn^A35T^, and subsequently named AD-LBV mice. The 3xTg-AD mice readily recapitulated Aβ and tau pathology, and after breeding with αSyn^A35T^ mice the resulting AD-LBV offspring exhibited accelerated cognitive deterioration, and enhanced accumulations of Aβ, αSyn, and tau pathology compared to 3xTg-AD or αSyn^A53T^ animals [199]. The AD-LBV mice exhibited increased levels of insoluble αSyn, phosphorylated αSyn (pS129), Aβ_1-42_, and tau at younger ages compared to the 3xTg-AD or αSyn-A53T animals [199].

A direct involvement of αSyn in the production of Aβ (Fig. 2) in humans could be suggested by results from Winslow and colleagues who described a novel molecular interaction between presenilin 1 (PS1), an important player in the proteolytic processing of the APP to yield Aβ [200], and αSyn [201]. Complexes of αSyn and PS1 where increased in AD and DLB patients with associated *PSEN1* mutations [201]. Another study showed that *PSEN1* mutations associated with AD and DLB aggravated phosphorylation and accumulation of αSyn [202].

Together the described results, although varying, from numerous studies of patient samples, animal and cell models propose that αSyn may play a leading role in a number of mechanisms and processes linked to AD. However, the heterogeneity in these processes have so far precluded the identification of exact mechanisms to be replicated in both in vivo and in vitro models. Such mechanisms could not only deepen our knowledge regarding the etiology of AD but also the pathogenesis of various synucleinopathies.

## Relevance of proposed α-synuclein propagation to pathophysiology of AD

As aforementioned, the neuropathological lesions of not only AD but also PD/DLB and amyotrophic lateral sclerosis (ALS) are known to spread through the brain in a rather specific pattern, which also serves as a basis for the neuropathological staging of disease severity [203]. The spreading of pathology was previously proposed to result from intra- and intercellular propagation of pathological seeds of tau, Aβ, αSyn and TDP-43 following a prion-like mechanism [204]. Specifically, the term ‘prion-like’ refers to unknown mechanisms in which toxic misfolded monomers or multimers have the innate ability to directly transmit disease to endogenous proteins by initiating an autocatalytic cycle of pathological misfolding and aggregation [203]. The most widely cited studies in support of the prion-like spread of LRP comes from post-mortem studies of two PD patient cohorts who received robust long-term intrastriatal grafts of human fetal midbrain tissue [205–208]. These studies showed that 1-5% of the grafted dopaminergic neurons exhibited detectable LRP 12-22 years post-transplantation (*n*=2 patients [205], *n*=1 patient [206], *n*=3 patients [207]), which increased to 11-12% after 24 years (*n*=1 patient [208]). A prion-like and αSyn-supported spread of AD pathology is potentially plausible as; i) it is widely known that in cell-free, cell and animal models, αSyn fibrils are readily able to catalyze the fibrillation of monomeric αSyn into aggregates of varying toxicity, ii) as mentioned herein, αSyn can also seed the aggregation of Aβ and tau, and iii) αSyn has the ability to spread to adjacent cells via secretion in exosomes [209], uptake via endocytosis [210] and cell-surface receptors like HSPGs, or even direct neuron-to-neuron [211, 212] or astrocyte-to-neuron [213] transfer via tunneling nanotubes (Fig. 2). Numerous aspects of a potential prion-like spread of αSyn in AD need to be considered including whether αSyn expression and secretion is altered and whether this is specific to certain brain areas. Importantly, the spatiotemporal spread of αSyn lesions in PD/DLB is nearly inverted to the spread of Aβ and tau pathology as observed in AD [203]. Hence the importance of specific neuronal populations and circuits to the potential spread of pathological seeds may differ between synucleinopathies like PD/DLB and AD. This notion can exemplified by the occurrence of LRP specifically in the amygdala of *PSEN1* mutation carriers [25]. Although the amygdala is heavily regulated by an inhibitory network of cells, it primarily responds to glutamate resulting in the secretion of neurotransmitters including acetylcholine, dopamine, serotonin and norepinephrine [214]. Furthermore, local concentrations of molecules like APOE and neurosin may alter the extracellular concentrations of αSyn, and thereby possibly influence the downstream spread of αSyn and AD pathogenesis. Detailed studies are needed in order to elucidate why the amygdala in particular is vulnerable to αSyn pathology.

## Concluding remarks

Numerous clinical studies have by now documented the presence of αSyn pathology in patients with AD, however, little attention has been devoted to αSyn as a potential target for AD disease-modification, prevention or cure. The accumulated data from human and murine cell cultures clearly suggests an involvement of αSyn in the GSK3β-mediated phosphorylation of tau which goes hand-in-hand with the strong correlations found between CSF levels of αSyn and tau in multiple neurodegenerative disorders including AD. Importantly, in vivo models also outline possible mechanisms in which αSyn-GSK3β activity could contribute to a pathological feedback loop in AD; Aβ could increase GSK3β activity which in turn can induce tau phosphorylation and αSyn production (and heterotrimeric complexes), all of which (Aβ, tau, and αSyn) can seed the aggregation of one another, leading to potentially more Aβ production, GSK3β activation (via more Aβ and αSyn), tau phosphorylation, cellular dysfunction and apoptosis. Importantly, recent data implies that the AD and DLB genetic risk factor *APOE*ε4 may be linked to processes connecting αSyn to the core AD pathological hallmarks, Aβ plaques and NFTs. Future studies facilitated by emerging techniques to image αSyn in the brain tissue ante-mortem will increase our understanding of the distribution of αSyn, αSyn pathology and its relevance to the pathogenesis and pathophysiology of AD.
